# Skin microbiota in health and disease: From sequencing to biology

**DOI:** 10.1111/1346-8138.15536

**Published:** 2020-08-17

**Authors:** Thomas H. A. Ederveen, Jos P. H. Smits, Jos Boekhorst, Joost Schalkwijk, Ellen H. van den Bogaard, Patrick L. J. M. Zeeuwen

**Affiliations:** ^1^ Center for Molecular and Biomolecular Informatics (CMBI) Radboud Institute for Molecular Life Sciences (RIMLS), Radboud University Medical Center (Radboudumc) Nijmegen The Netherlands; ^2^ Department of Dermatology RIMLS, Radboudumc Nijmegen The Netherlands; ^3^ NIZO Ede The Netherlands

**Keywords:** bioinformatics, cutaneous diseases, microbiomics, next‐generation sequencing, skin microbiota

## Abstract

Microbiota live in a closely regulated interaction with their environment, and vice versa. The presence and absence of microbial entities is greatly influenced by features of the niche in which they thrive. Characteristic of this phenomenon is that different human skin sites harbor niche‐specific communities of microbes. Microbial diversity is considerable, and the current challenge lies in determining which microbes and (corresponding) functionality are of importance to a given ecological niche. Furthermore, as there is increasing evidence of microbial involvement in health and disease, the need arises to fundamentally understand microbiome processes for application in health care, nutrition and personal care products (e.g. diet, cosmetics, probiotics). This review provides a current overview of state‐of‐the‐art sequencing‐based techniques and corresponding data analysis methodology for profiling of complex microbial communities. Furthermore, we also summarize the existing knowledge regarding cutaneous microbiota and their human host for a wide range of skin diseases.

## Microorganisms are Omnipresent

Consortia of microbes are found in many niches of the earth, like on various sites of animals and plants, in soil, in water and in the atmosphere, but also in industrial fermentations and biofilms. In this review, we will focus on the human skin microbiota (mainly on bacteria), their currently known relevance in health and disease, and provide an overview of main sequencing‐based methods and bioinformatic tools to measure them. The relevance of bacteria for human life becomes clear by looking at the numbers, as bacteria colonize our body sites with a ratio of 1.3:1 bacterium to human cell.^1^ Even if they did not evoke an effect on bodily processes – but which many of them do – their sheer number in members, genes and variation relative to their host alone, make them interesting subjects of research.

## Next‐Generation Sequencing of Microbiota

**Figure 1:**
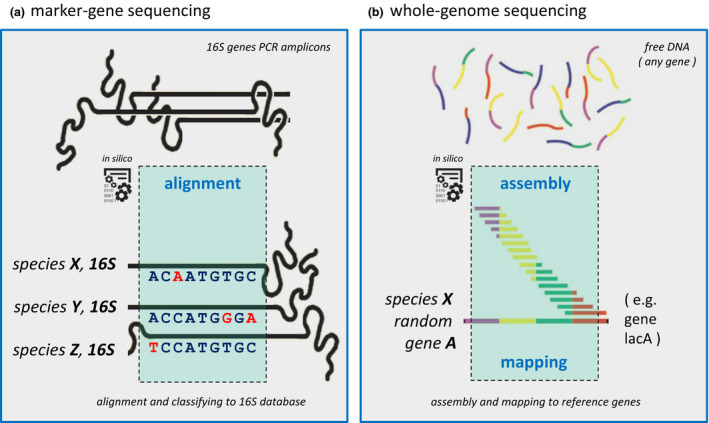
Principal microbiota sequencing approaches. We roughly distinguish two main applications for next‐generation sequencing (NGS) of microbial communities: marker‐gene sequencing (MGS) for metataxonomics and whole‐genome sequencing (WGS) for metagenomics. (a) In the MGS example, 16S is selected as marker gene, which is extracted from a mixed microbial population by polymerase chain reaction (PCR, not shown), and sequenced by NGS techniques. After MGS sequencing, reads (±500 bp) are aligned, and based on informative positional differences in the 16S gene, known reference microbiota can be assigned, or novel taxonomies can be inferred. With WGS, one can extract genomic potential and function information, in contrast with MGS, as with the latter, one can only extract taxonomic information. (b) In the WGS example, typically small sequences (100–150 bp), derived randomly from the full genomic content (i.e. all genes present, not focusing only on 16S) of a mixed microbial population, are assembled into genes of all microbiota present.

Currently, next‐generation sequencing (NGS) advances allow for high‐throughput, massively parallel and deep sequencing of DNA samples, thereby dismissing the need for vector‐based cloning of sequences. NGS has given an enormous boost to the field of genomics, microbiomics and bioinformatics, amongst others, mainly due to its substantially reduced sequencing costs and ultra‐high‐throughput application. Bacterial consortia are currently mostly analyzed either by marker gene sequencing (MGS) metataxonomics or by whole‐genome sequencing (WGS, in this review also referred to as “shotgun”) metagenomics (Fig. 1). Metagenomics is defined as the study of the collection of genomes and genes from the members of a microbiome.^2^ It is not to be confused with MGS initiatives such as bacterial 16S (or its fungal counterpart internal transcribed spacer) amplicon sequencing, which are better described as metataxonomics: the high‐throughput identification, classification and naming of microbiota.

## MGS

**Figure 2:**
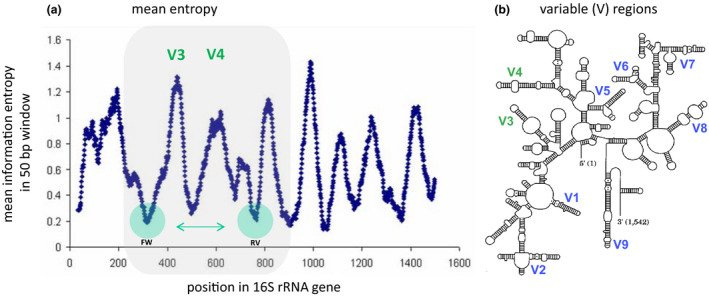
16S rRNA marker gene characteristics. The 16S rRNA gene in bacteria is widely used for metataxonomics. Between different clades of phylogenetically‐related bacteria, this gene varies strongly in terms of conservation and variation, as shown in the left panel by the consecutive peaks and valleys in that graph. (a) Visualization of the mean information entropy for each position of the 16S gene (±1.5 kbp in length), based on all known 16S genes present in the Ribosomal Database Project. One can see peaks (strong variation) and valleys (strong conservation) in different regions of the 16S gene sequence, regions which can be used for taxonomic discrimination and primer design, respectively. In this example, we observe nine peaks (variable regions), of which V3 and V6 show the largest peaks and deepest valleys. Therefore, the gene region from V3 up to V6 is very suitable for primer design and marker‐gene sequencing. Nonetheless, as most currently applied short‐read next‐generation sequencing applications are not able to sequence for more than 500 bp, like for Illumina MiSeq, one has to choose for a shorter region length, such as for V1–V2, or for V3–V4 as illustrated here. Figure adapted from Andersson *et al*.^81^. (b) The nine different variable regions, structurally visualized over the full length of the 16S gene. The locations of V3 and V4 are indicated in green.

There are many types of marker genes that can be utilized for bacterial MGS, but the gene which is most widely chosen for this purpose is the universal 16S rRNA gene (16S, in short). In this section we will therefore focus on 16S, but the principles and application of alternative marker genes are similar; for example, see Scholz *et al*.^3^ who use an alternative gene to specifically type *Cutibacterium acnes* species in high resolution. For more information on the use of alternative bacterial marker genes, we refer to our recently published generic workflow for discovery and analysis of single‐locus sequence typing (SLST) marker genes.^4^ Please note that a marker gene in the context of bacterial MGS should not be confused with marker genes in the context of molecular cloning, where the term “marker gene” has been adopted for genes used to indicate successful genome editing. 16S MGS focuses on the 16S rRNA genes present in all prokaryotes and archaea. In theory, the 16S rRNA gene can be targeted by universal polymerase chain reaction (PCR) primers, and the technique does therefore not require bacterial reference genomes for analysis. However, for classifying 16S sequencing reads, prior knowledge in the form of 16S rRNA gene databases with corresponding taxonomy information is required. Most notable, the Ribosomal Database Project,^5^ Greengenes^6^ and SILVA^7^ are well‐established examples of such databases. 16S allows for confident profiling of bacteria down to the genus level. The process of analyzing 16S sequencing reads usually involves clustering of sequencing reads in operational taxonomic units (OTU), where they are classified (Fig. 1a). Although OTU picking is very commonly applied when analyzing 16S data, there are some non‐OTU‐based alternatives for analyzing microbiomes, such as by oligotyping or by using exact sequence variants as suggested in a recent review by Knight *et al*.^8^ Oligotyping allows for discriminating between closely related but distinct taxa by looking at position‐specific information in 16S rRNA sequences. In practice, for 16S, there is a delicate trade‐off between taxonomic sensitivity and resolution (specificity) in terms of microbiota classification (the “sensitivity‐to‐classification” problem). Hence, determining which 16S primers to use is crucial (Fig. 2a). The 16S rRNA gene has multiple alternating conserved and variable (V1–V9) regions, with a total length of roughly 1.5 kbp (Fig. 2b). For application in sequencing, the longer the 16S sequencing read the more confidently microbial classification can be performed. Therefore, depending on the sequencing platform, one best selects those V‐regions that maximize potential of their sequencing platform with regard to read length. In case of low input DNA for 16S amplification by PCR, which is a notorious problem for skin samples, one has the option to choose a nested PCR (or two‐step PCR). First performing a PCR to amplify a large 16S regions such as V3–V6, and a second round of PCR to amplify another smaller region within the large region, such as V3–V4, can be beneficial to boost MGS amplicon yield as input for sequencing. Furthermore, every 16S primer (combination) has its pros and cons when it comes to sensitivity and specificity to different bacterial families (genera).^9^ Primer choice also depends on the target niche of interest, such as for gut, skin or oral samples, which therefore is additionally motivated by bacteria typically present on those sites.^10^ To analyze MGS data, many different methods and bioinformatic tools are available. OTU methods like QIIME,^11^ which by default uses the UCLUST OTU clustering algorithm,^12^ and Mothur,^13^ based on average linkage OTU clustering, are widely used and commonly accepted.^14^, ^15^

## Metagenome Sequencing

Shotgun metagenomics, in contrast to MGS, does not suffer from the aforementioned sensitivity‐to‐classification problem as it sequences all (free) DNA present in a sample (Fig. 1b).^16^ The major advantage of shotgun over MGS is that it provides insight into gene and metabolic function potential of a sample based on its metagenome. This allows for pathway analysis and even mining for virulence factors, antibiotic resistance, (pathogen) lineage‐specific markers or novel enzymes for catalysis of reactions in food, pharmaceutical or industrial processes. Alternatively, complete bacterial genomes can be extracted from shotgun samples if the metagenomics data is assembled, provided that the genomes are present in high enough numbers to achieve a minimum feasible reads‐to‐genome coverage to allow for confident assembly.^17^ Furthermore, apart from bacteria and fungi, shotgun enables retrieval of DNA sequences from viruses (i.e. non‐RNA), bacteriophages, eukaryotes and even host cells. The latter can be problematic in terms of contamination. In cutaneous microbiome studies, this can be a notorious problem, as skin has a low microbe‐to‐host ratio resulting in few bacteria and a large number of dead host cells that are easily taken along upon skin sampling.^16^ Therefore, minimizing host DNA contamination during microbial DNA sampling is of importance to yield high‐quality sequencing data. For skin, depending on the specific research question at hand, one may want to minimize mechanical disruption of the skin by taking skin (wet) swabs, in contrast to the skin scraping alternative. Instead, if measuring microbiota from deeper skin layers is desirable (e.g. to inspect deeper layers or to minimize measuring transient microbiota), one may want to choose from methods that use skin scraping by a sterile scalpel or by tape stripping. Unfortunately, shotgun needs much more microbial DNA input than MGS, is very labor intensive for both personnel and computation, and should therefore ideally be applied only when there is a specific hypothesis to be tested: for example, is a particular (new) functionality such as antibiotic resistance or virulence factors present, and is it linked to certain bacteria? Typically, shotgun metagenomic dataset resolution allow for classification to the level of genus/species with tools such as MetaPhlAn,^18^ MEGAN,^19^ AMPHORA^20^ and mOTUs,^21^ or even down to subspecies level with tools such as ConStrains,^22^ PanPhlAn^23^ and StrainPhlAn.^24^ However, these methods can be used confidently only for (sub)species with an overall relative abundance of 1% or above. With shotgun, it is in principle possible to determine the presence of entities close to the strain level, but this requires complex methods and typically large datasets are involved.

Recently, the concept of multi‐locus sequence typing has been adopted for high‐resolution bacterial classification in NGS efforts by initiatives such as PathoScope^25^ and PhyloPhlAn^26^ that provide sets of representative marker genes and algorithms for phylogenetic inference of sequencing reads. Likewise, metagenomic analysis methods such as AMPHORA, ConStrains, MetaPhlAn and StrainPhlAn effectively apply in high throughput the concept of single nucleotide variant patterns in marker genes. For pathway reconstruction and analysis of potential functions of microbiota, various bioinformatic tools exist and can be adopted, most notably MG‐RAST^27^ and HUMAnN (includes ChocoPhlAn),^18^ although some of the aforementioned tools for microbial classification also offer a function analysis to some extent. Noteworthy, methods such as PICRUSt,^28^ PanFP^29^ and Tax4Fun^30^ are alternatives to shotgun in order to yield information about microbial function from 16S sequencing data. It deduces the presence of bacteria based on 16S reads, and infers from this information the total metabolic potential of a microbiome with a taxon‐to‐function reference database. Albeit conceivably inferior to shotgun metagenomics, it does provide a coarse‐grained overview of the metabolic capacity of a microbiome. In opposite fashion, one is able to “mine” 16S rRNA or other marker genes from shotgun data, which could be interesting for a quick screen, or when there is specific interest in one particular phylogenetic clade or (associated) function.

Microbial strains are the most specific source of information with regard to metabolic and regulatory potential of a microbiome, and follow‐up experiments are straight‐forward to perform with (combinations of) microbial strains. Hence, pinpointing the right candidate strains from studies with access to shotgun data is crucial; however, getting enough (DNA) material for WGS can be a challenge for some biological sources like low microbial density skin sites. Nevertheless, new WGS techniques with larger data output are emerging rapidly, and altogether the microbiome community seems to be on the verge of a shift from MGS to shotgun as a general application for profiling of microbiota.

## Cutaneous Microbiome

Simply put, the human skin is a physical barrier of the body that has one main purpose: to keep the inside in, and the outside out. In addition, the skin functions as an immunological barrier with processes like microbial colonization resistance by the skin microbiome, and host immune sensing and surveillance.^31^ The skin is anatomically comprised of several layers with different cells and properties. The epidermis largely (>90%) consists of keratinocytes, an epithelial cell type with barrier and host defense functions, and can be divided into different strata. The stratum basale is the germinative layer from which the cells migrate to the skin surface in approximately 20 days. Ultimately, in the last living cell layer of the epidermis (stratum granulosum), cells excrete large amounts of lipids, form a cross‐linked cell envelope and lose their nuclei. These enucleated cornified envelopes form the stratum corneum. The stratum corneum is where most of the skin microorganisms reside, and the deeper into the stratum corneum the less microbes are present.^16^ The estimated microbial density on the total skin surface (from all body parts combined) is very low compared with the entire large intestine, but it is comparable with quantities found in the complete small intestine, with estimates of 10^11^ bacterial cells in total.^1^ Note that this calculation does not take into account microbiota from the deeper layers, only from the skin surface. Normally, the epidermal layers beneath the stratum corneum and the dermis are considered sterile with the exception of sweat and sebaceous glands and hair follicles, which are also colonized by microbes.^32^

Microbial make up of skin niches is highly dependent on characteristics based on skin type and location. We distinguish three main physiological skin sites: (i) oily/sebaceous skin sites such as the forehead, the upper back and the skin behind the ear; (ii) dry skin sites such as the forearm and lower back; and (iii) moist skin sites such as the armpits, backs of knees, nostrils and groin. But also acidity (pH), salt content and temperature of the microenvironments are important drivers for microbial inhabitants. Despite site‐to‐site compositional variation, common skin commensals typically found on humans are the genera *Corynebacterium*, *Cutibacterium*, *Staphylococcus*, *Micrococcus*, *Actinomyces*, *Streptococcus* and *Prevotella*.^16^, ^33^ Even though interindividual variation between healthy volunteers is high, microbial communities are largely stable over time, despite exposure of the skin to the external environment.^34^, ^35^ However, mechanical disruption of the skin barrier results in temporary change in microbial composition up until recovery of the skin.^16^

Both the stratum corneum and the differentiated cell layers of the epidermis are essential to control microbial invasion by providing a physical barrier and an antimicrobial protein (AMP) shield. Keratinocytes express many AMP in order to control skin microbiota colonization and infection. Notable examples are psoriasin (S100A7) and human β‐defensins (hBD‐2) that target Gram‐negative bacteria such as *Escherichia coli* and *Pseudomonas aeruginosa*. Other important skin AMP are SKALP/elafin, hBD‐3, SLPI, calprotectin (complex of S100A8/S100A9), LL37 (CAMP), lysozyme, RNase‐7, and the recently reported group of late cornified envelope (LCE) proteins.^36^, ^37^, ^38^, ^39^, ^40^, ^41^, ^42^, ^43^

## Cutaneous Microbiome and Inflammatory Skin Conditions

Over the past years, many studies have been conducted on lesional skin of common skin diseases to identify which species are most abundant and whether this microbial composition differs from healthy skin. Although bacteria are normally presented as commensal microbes, in certain situations (e.g. when the skin barrier is compromised or the immune system is failing), some of these bacterial species can become opportunistic pathogens that give rise to infections. Perturbations of “normal” microbial communities where homeostasis between the host and its microbiota are disturbed is called “dysbiosis”. Many common skin conditions are associated with a dysbiotic state of the skin microbiota, with the most research conducted on the inflammatory skin disease atopic dermatitis (AD).^44^

## AD

Atopic dermatitis is a common chronic skin condition mostly affecting infants, and is characterized by pruritic inflammatory skin patches. The risk factors for development of AD are multifactorial, ranging from environmental to genetic susceptibility factors. The disease pathophysiology is complex, with impaired epidermal barrier function, T‐helper (Th)2 cell‐mediated inflammation and neuroimmune interactions which have been reviewed extensively by Weidinger *et al*.^45^ In addition to the aforementioned contributing factors to AD development, dysbiosis of the skin microbiota has been described already in the 1970s, where it was shown that *Staphylococcus aureus* is overrepresented on the skin of AD patients. In addition, whereas the healthy cutaneous microbiota composition is diverse, the composition of lesional AD skin is far less diverse and consists mainly of *S. aureus*, a bacterium that in healthy situations can be a commensal on the skin and mucous membranes.^46^ *S. aureus* can become an opportunistic pathogen with a plethora of weaponry to make sure it adheres to the skin,^47^ weakens the skin barrier^48^ and triggers the immune system,^49^ which eventually exacerbates the cutaneous inflammation (as reviewed recently by Paller *et al*.).^50^ Interestingly, a study by Chng *et al*.^51^ recently showed that the bacterial composition of AD‐prone, non‐flare skin is more diverse than the composition of healthy skin. An enriched abundance of *Streptococcus*, *Gemella* and *Haemophilus* species and decreased abundance of *Dermacoccus* species was presented on AD‐prone, non‐flare skin versus normal healthy skin. Whereas it is known that *Staphylococcus* species can invoke a strong cytokine and chemokine response in order to induce inflammation, the bacteria present in AD flare‐prone skin seem to mute this response, suggesting a protective role for these bacteria.^51^ Recently, a large cohort study was presented that combined skin microbiomes (AD and psoriasis) and associated host transcriptomes.^52^ Unique gene profiles were identified that distinguish healthy from inflamed skin, and colonization of AD skin by *S. aureus* was associated with dysregulation of genes involved in epithelial barrier function, immune activation and tryptophan metabolism.^52^ Furthermore, lesional AD skin could be more amenable for colonization by *S. aureus* because of an imbalanced antimicrobial response. Where healthy skin expresses many different antimicrobial molecules (e.g. β‐defensins, cathelicidins, free fatty acids and reactive oxygen species), the skin of AD patients has been shown to have a reduced expression of defensins and cathelicidins.^53^ This could partly be explained by Th2 cytokines which have been shown to suppress the expression of such AMP.^54^, ^55^ Recently, it was reported that coal tar treatment of AD lesions induces AMP production via canonical aryl hydrocarbon receptor (AHR) signaling.^56^ By application of a novel SLST MGS method,^4^ a shift in microbiota composition toward that of healthy controls was observed, which suggests that restoring AMP levels in AD skin via AHR‐dependent transcription regulation can be beneficial by creating an (anti)microbial milieu that is less prone to infection and inflammation. However, more studies are required to address the causal relation between AMP profiles and their effect on the cutaneous microbiota composition of AD skin. Furthermore, colonization resistance has been described in AD, where certain coagulase‐negative *Staphylococcus* strains hamper the growth of *S. aureus* by expressing antimicrobial peptides.^57^ Our group also showed that the skin microbiota of filaggrin (*FLG*)‐deficient patients (*FLG^–/–^*), compared with healthy controls (*FLG^+/+^*), contain a lower relative abundance of Gram‐positive anaerobic cocci (GPAC). This is thought to be the result of the absence of FLG and its degradation products, namely natural moisturizing factors which GPAC use as a carbon source.^58^ In addition, we showed that GPAC microbes, such as *Finegoldia* spp., can quickly induce the expression of the antimicrobial proteins hBD‐2, hBD‐3 and LL37 by keratinocytes.^58^ In the absence of GPAC, the antimicrobial response may be hampered or delayed in AD patients with *FLG* mutations, which can favor *S. aureus* colonization and infection.

## Psoriasis

Research on the psoriasis microbiome has shown that microbiota composition differs widely between healthy and psoriatic skin; however, the results between the studies are contradictory. Firmicutes were reported to be overrepresented while Actinobacteria were underrepresented in one study,^59^ and another study claimed that *Staphylococcus* species were overrepresented in an overall less diverse microbiome.^60^ The next study claimed a decrease in the relative abundance of *Cutibacterium* and *Staphylococcus* species, and an increase in *Corynebacterium*.^61^ Overall, due to these conflicting results, it is safe to conclude that a general psoriasis microbiome has not been deciphered yet, or perhaps a characteristic psoriasis‐like microbiome does not exist and the current observations are best explained by strong interindividual variation within the psoriasis phenotype.^62^, ^63^

## Acne Vulgaris

Acne vulgaris is a chronic skin disease of the pilosebaceous unit, which are sebaceous glands that are connected to hair follicles, and important for the secretion of sebum. Although *C. acnes* is highly common in healthy adults, its presence and formation of biofilms are associated with acne vulgaris.^64^ The anaerobic and lipid‐rich milieu of the sebaceous gland provides the optimal environment for *C. acnes* to thrive, especially when the follicle shaft is blocked. Recent metagenomic studies on several *C. acnes* strains have shown that genetic differences can possibly explain why some *C. acnes* strains act as commensals and other strains act as pathogens.^65^ Furthermore, it was shown that skin microbiome differences relate to the grade of acne vulgaris.^66^ These studies underline the importance and added value of performing metagenomic studies to go up to strain‐level depth.

## Rosacea

Characteristics of rosacea, a common inflammatory condition of the facial skin, are facial flushing, redness, papules and pustules of which the pathogenesis is largely unknown.^67^ Evidence of rosacea‐associated microorganisms has been shown for *Staphylococcus epidermidis*, *Helicobacter pylori* and *Chlamydophila pneumonia*. Colonization with *Demodex folliculorum* mites (and the microbiota they carry) also positively correlates with disease severity.^68^ Furthermore, 16S rRNA gene sequencing showed that different subtypes of rosacea harbor *Demodex* mites with different microbiota.^69^

## Seborrheic Dermatitis

Seborrheic dermatitis (SD) and dandruff are chronic skin conditions that are often displayed on skin that is rich in sebaceous glands, like the upper back, nose and scalp. It is thought that both conditions are within the spectrum of the same disease with a different severity and location.^70^ The pathophysiology of both conditions is not understood completely, but it has been shown that fungal colonization is a predisposing factor. *Malassezia* fungi *Malassezia globosa* and *Malassezia restricta* were identified as the predominant fungi on both normal skin and the scalp of SD and dandruff patients, and the amount of fungi was shown to correlate with disease severity.^71^, ^72^

## Skin Wounds and Cutaneous Infections

Chronic wounds, such as diabetic foot ulcers, postsurgical wounds or decubitus ulcers, are an ideal place for bacterial overgrowth, and frequently these ulcers show impaired cutaneous wound healing.^73^ Similar to acne vulgaris, biofilms are regularly formed by monocolonization of one or a few bacterial species, thereby further fostering pathogenic growth and making these wounds particularly difficult to heal.^74^ Bacterial species from the *Staphylococcus* genus (e.g. *S. aureus* and *S. epidermidis*) and *Pseudomonas* (e.g. *P. aeruginosa*) genus are often identified in chronic wounds,^75^ just like bacteria from other anaerobic genera (e.g. *Finegoldia*, *Peptoniphilus*, *Peptostreptococcus*).^76^ Acute cutaneous infections (e.g. resulting from burn wounds) frequently display an altered bacterial composition that is characterized by elevated abundances of thermophilic bacteria like *Aeribacillus*, *Caldalkalibacillus*, *Nesterenkonia* and *Halomonas*, and by a decrease in the relative abundance of skin commensals such as *Cutibacterium* and *Corynebacterium*.^77^ It is speculated that these thermophiles are introduced during debridement procedures, or that their increase is the result of the disruption of the skin barrier, thereby supplying nutrients to these bacteria. More clinical observations are needed to further validate these theories.^78^

## Concluding Remarks

**Figure 3:**
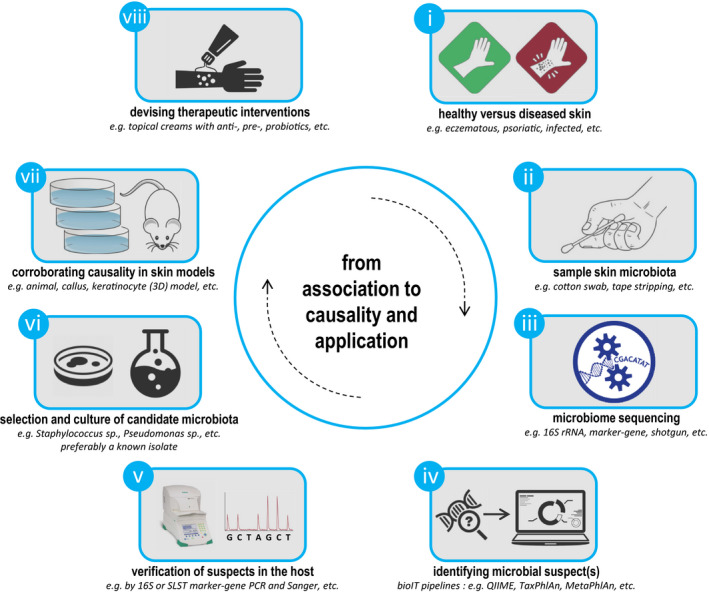
Studies do not stop at association. Schematic typical workflow/study approach in order to potentially go from association to causality, with specific focus on skin (micro)biology. (i) Healthy versus affected skin is evaluated, with suspected involvement of the microbiome as causative driver. (ii) Skin samples are collected through standard protocols, and (iii) are sequenced by a suitable platform depending on research question and study budget. (iv) Microbial suspects are identified by available data analysis pipelines, and (v) their specific presence and (differential) abundance are validated in the host by alternative (conventional) methods. Thereafter, (vi) candidates are selected and cultured for (vii) corroboration of microbiota‐associated effects of initial study findings by relevant *in vitro* or *in vivo* (disease) models. Finally, if applicable, (viii) functional applications could potentially be devised from study findings (e.g. organisms, proteins, compounds, protease inhibitors), ultimately leading to novel therapeutic interventions for treatment of skin disease. PCR, polymerase chain reaction; SLST, single‐locus sequence typing.

In conclusion, commensal bacteria can play a pathogenic role under certain conditions, which further underlines the significance of studying skin diseases in the context of host genetics, immune responses, skin barrier function and the complete microbiome. Furthermore, microbe–microbe interactions can play an additional role in the pathogenesis of skin conditions. For identifying (skin) microbiota, MGS is currently still the most accessible approach for most applications, when it comes down to technical and methodological complexity. Nevertheless, we foresee that WGS will take the stage in the following years, which will enable functional analysis and a higher taxonomical resolution profiling of the abundant microbial fractions. Importantly, it should be pointed out that microbiome sequencing‐based studies can in principle only find correlations and associations, and cannot directly elucidate causal relations. In addition, we observe that the majority of microbiome studies still report association‐based results, whereas validation of associations and a mechanistic understanding thereof is what we should ideally strive for (Fig. 3). This will require the use of models such as *in vitro* (3‐D) skin systems^79^ or (germ‐free) animal models, where the effect of specific candidate microbial isolates or a synthetic consortium of microbes (minimal microbiome) on the model can be tested (hence, microbial candidates identified in pilot/initial sequencing‐based studies). For *in vivo* modulation of cutaneous microbiota as a tool for keeping a healthy skin microbiome, skin microbiota transplantation is finding its way into research applications, as are pre‐ and probiotics for topical application.^80^ In the end, our goal should be functional applications that could potentially be devised from our microbiome study findings (e.g. organisms, proteins, compounds, protease inhibitors), ultimately leading to novel therapeutic interventions for treatment of skin disease.

## Conflict of Interest

None declared.
